# Validation of New and Existing Decision Rules for the Estimation of Beat-to-Beat Pulse Transit Time

**DOI:** 10.1155/2015/306934

**Published:** 2015-03-02

**Authors:** Xiaolin Zhou, Rongchao Peng, Hongxia Ding, Ningling Zhang, Pan Li

**Affiliations:** ^1^Institute of Biomedical and Health Engineering, Shenzhen Institutes of Advanced Technology, Chinese Academy of Sciences, Xili Nanshan, Shenzhen 518055, China; ^2^The Key Laboratory for Health Informatics of the Chinese Academy of Sciences at Shenzhen Institutes of Advanced Technology, Shenzhen 518055, China

## Abstract

Pulse transit time (PTT) is a pivotal marker of vascular stiffness. Because the actual PTT duration in vivo is unknown and the complicated variation in waveform may occur, the robust determination of characteristic point is still a very difficult task in the PTT estimation. Our objective is to devise a method for real-time estimation of PTT duration in pulse wave. It has an ability to reduce the interference caused by both high- and low-frequency noise. The reproducibility and performance of these methods are assessed on both artificial and clinical pulse data. Artificial data are generated to investigate the reproducibility with various signal-to-noise ratios. For all artificial data, the mean biases obtained from all methods are less than 1 ms; collectively, this newly proposed method has minimum standard deviation (SD, <1 ms). A set of data from 33 participants together with the synchronously recorded continuous blood pressure data are used to investigate the correlation coefficient (CC). The statistical analysis shows that our method has maximum values of mean CC (0.5231), sum of CCs (17.26), and median CC (0.5695) and has the minimum SD of CCs (0.1943). Overall, the test results in this study indicate that the newly developed method has advantages over traditional decision rules for the PTT measurement.

## 1. Background

Arterial pulse-wave velocity (PWV) is a valuable surrogate marker for vascular stiffness. Increased arterial stiffness, as assessed noninvasively by the measurement of PWV, is associated with increased risk of clinical cardiovascular disease (CVD) events [1–4]. In a population-based study among healthy participants, subjects with increasing PWV were 2.45 times more likely to develop CVDs during a mean follow-up period of 4.1 years [5]. It is well delineated that the relative risk of follow-up CVD events increases with increasing level of PWV. Indeed, the higher aortic PWV was associated with a 48% increase in CVD risk [6]. The arterial wall thickens and the arteries get stiffer with aging, whilst elevated blood pressure (BP) may occur as a result of stiffer arteries. The preclinical CVD events should be intervened in the early stage. As such, given the independent predictive value of PWV, identifying strategies that might be used in reducing the incidence of events led by the acceleration of the arterial aging process may have certain importance in prevention of CVDs [7].

PWV is calculated as the distance traveled by a pulse wave divided by the time (also referred to as the pulse transit time (PTT)) for the wave to travel that distance. For each cardiac circle, the PTT can be manually defined in two ways: (a) the time interval between the R-wave peak of a QRS complex in electrocardiogram and some fiducial point of the subsequent arrival of the pulse wave at the finger; (b) the time delay of two pulse waveforms which are detected from separate arterial sites. For the first approach, the cardiac preejection period might be a confounding factor for robust PTT estimation [8]. Over the past decades, a number of methods for measuring the PTT have been proposed and most vary in the way the fiducial point on pulse waveform is determined [9–21]. In a broad sense, these methods for determining the fiducial point can be categorized into threshold methods, differential methods, or transect intersection methods. The accuracies of current methods for the PTT estimation have been limited by the difficulties encountered in the signals with high-level noise [22]. The straightforward threshold-based methods are sensitive to low-frequency baseline modulations (e.g., baseline drifting and respiratory fluctuations). The differential methods are susceptible to wide frequency-band noises, such as white Gaussian noise, since the differential filters (i.e., the forward/backward and central derivatives) lack for the capability of effectually suppressing the high-frequency noise. Regarding the transect intersection methods, because the realization process involves reference to the minimum point of the pulse wave which is sensitive to the fluctuations around the local area as well as the distinctive point which is generally determined on the differential signals, they are vulnerable to both low- and high-frequency noises. Determination of the quantitative relevance of PTT and BP is time-consuming and cumbersome in most clinical situations [23]. In addition, the issues of accuracy and reproducibility of various decision rules for PTT measurement have not been well explored.

The research described herein develops a new method which is based on the first-order, central derivative and the centroid strategy for the determination of the fiducial point in PTT measurement. The new method should have the ability to reduce both baseline drifting and respiratory fluctuations and, by using the centroid strategy over many optimally selected points of differential signals to determine the fiducial point, it could markedly reduce the interference caused by high-frequency noise. The performances of the proposed method and existing methods are evaluated in detail with artificial and clinical pulse data in terms of the reproducibility and correlation coefficient (CC). Using artificial pulse data provides the following benefits: (A) the locations of feature points as defined by different methods in each pulse cycle can be specified exactly as the “true” references, which indicate that the reproducibilities are equal to 100% for all of the test methods; (B) by mixing a certain intensity of artificial noise into the generated pulse data, the signal-to-noise ratios (SNR) can be reset to any level, which reveals that the reproducibilities of different methods may be significantly different for artificial pulse data with various SNR levels: the higher reproducibility means the better performance. Accurate estimation of PTT recently received heat debate concerning the practical situation of PTT measurement: we could not obtain the true PTT duration in vivo [21, 24–26]. Therefore, a set of long-term clinical pulse data (acquired from 33 volunteers) are also tested to confirm the CC between the beat-to-beat PTT changes and the corresponding beat-to-beat BP variations which are synchronously recorded from the authorized medical device.

## 2. Methods

### 2.1. Moens-Korteweg Equation

**Figure 1:**
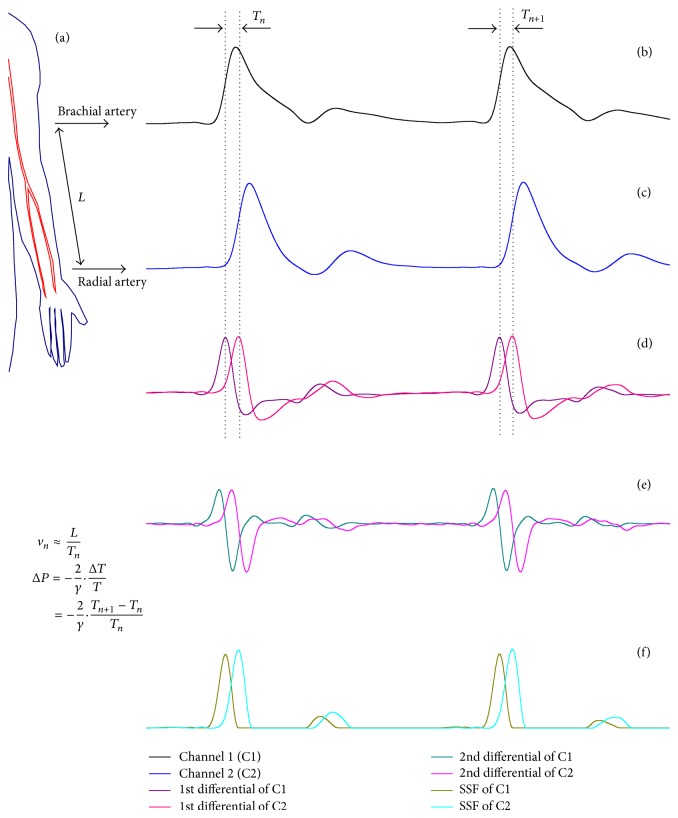
The PTT estimation using simultaneous noninvasive measurements of pressure waveform in the respective arm arteries. (a) Schema of pulse signals acquisition. (b) Brachial arterial pressure signal. (c) Radial arterial pressure signal. (d) Differential signals of brachial/radial pulse signals using the first-order derivative. (e) Differential signals of brachial/radial pulse signals using the second-order derivative. (f) Coefficients of slope sum function of two channel signals. In this example, the PTT is calculated by the first-order differential method, and *n* is the beat number. See text for abbreviation.

The Moens-Korteweg equation is a classic model for quantitatively identifying the relationship between the PWV and character traits of vessel (and contained blood within the vessel), which can be expressed by

(1) $$\begin{array}{c} v=\frac{L}{T}=\sqrt{\frac{hE}{r\rho }}, \end{array}$$

where *h* and *E* are the arterial wall thickness and Young's modulus, respectively. *ρ* and *r* are the density of blood and inside radius of vessel, respectively. *L* is the distance between two arterial sites through which a pulse wave passes over the corresponding transmit time *T* (i.e., PTT), and *v* is the PWV. See Figure 1 for clarification.

It has been commonly recognized that as the intravascular pressure *P* increases, Young's modulus *E* increases exponentially. This relationship is of the following form:

(2) $$\begin{array}{c} E=E_{0}e^{\gamma P}, \end{array}$$

where *E*_0_ is the zero-pressure modulus. *γ* is a constant that depends on the particular vessel. Substituting (2) into the Moens-Korteweg equation (1) and after a simple rearrangement, we therefore obtain [27]

(3) $$\begin{array}{c} P=\frac{1}{\gamma }\left[\ln\left(\frac{\rho rL^{2}}{hE_{0}}\right)-2\ln T\right]. \end{array}$$

As an approximation, we first assume that parameters *r*, *ρ*, *E*_0_, and *h* in (3) are nearly constants during a short period of time and then take the derivative of both sides of (3) with respect to *T*, which results in [27]

(4) $$\begin{array}{c} \Delta P\approx \frac{2}{\gamma }\cdot \left(-\frac{\Delta T}{T}\right). \end{array}$$

An increase in BP causes an increase in PWV and accordingly a decrease in the PTT. On the basis of the previously established postulate, from (4), it is clear that the increment of BP (Δ*P*) is approximately proportional to the relative increment of PTT (−Δ*T*/*T*). We will apply the correlation between Δ*P* and −Δ*T*/*T*, as shown in (4), as a performance metrics of PTT estimation in vivo tests.

### 2.2. Test Data Materials

Both artificial and clinical pulse wave data were collected for investigation. From the perspective of digital signal acquisition, the sampling frequency was set to 5000 Hz for both types of signals to accommodate the need for the reliable PTT estimation.

The original artificial pulse data were simulated by a previous research group [24], and these data are publicly available at [28]. The authors generated the data based on a validated, nonlinear, and one-dimensional mathematical model of distributed arterial tree [29]. We employed these original artificial data to construct long-term pulse data. To investigate the performances of the herein candidate methods in a various-level noise environment, the theoretic artificial pulse signals were mixed with different intensities of artificial noises including white Gaussian noise and the artificial respiratory signals simulated by a cosine function.

A total of 33 subjects (including 6 subjects having a history of hypertension, 6 subjects with hypotension, and 5 subjects with the presence of Sinus bradycardia; 16 were male and 17 were female) were randomly recruited to the clinical study. Their average age was 29.7 ± 8.7 years (range: 23–62 years). Average height and weight were 166.9 ± 8.4 cm (range: 150–185 cm) and 58.3 ± 10.7 kg (range: 40–83 kg), respectively; and their mean body mass index was 20.8 ± 2.8 kg/m^2^ (range: 16.9–28.7 kg/m^2^). The experiment was approved by the SIAT Research Ethics Committee (IRB number: SIAT-IRB-140215-H0040), and the written consent was obtained from all participants.

The experiment was performed in a quiet laboratory environment. Studies were conducted with all volunteers lying still in their supine position for about one and a half hours each. Figure 1(a) depicts the sketch of experimental setup; for each volunteer, the noninvasive brachial and radial pressure waveforms were acquired by two circular piezoelectric transducers placed over the left arm and held in place by two straps around the brachial and radial sites, respectively. To reduce the error caused by analog-to-digital converter quantization (i.e., to maintain high signal resolution), the 4-channel, 24-bit resolution NI USB-9239 data acquisition board (National Instruments, Austin, TX, USA) was used for pulse data acquisition. The continuous Finapres BP machine (Finapres Medical Systems B.V., The Netherlands) was employed to simultaneously measure the beat-to-beat BP waveforms by an inflating cuff dynamically fitting around the middle finger at the right hand. To ensure synchronization of the pulse waveforms acquired by the USB-9239 module and the continuous BP waveforms detected by the Finapres device, both of them were recorded in the same computer. The involved software of Finapres device automatically constructed the brachial BP data (systolic/diastolic/mean BP) from the finger BP data. The systolic BP data was used for evaluation in this study.

### 2.3. Digital Signal Processing

#### 2.3.1. Preprocessing

**Figure 2:**
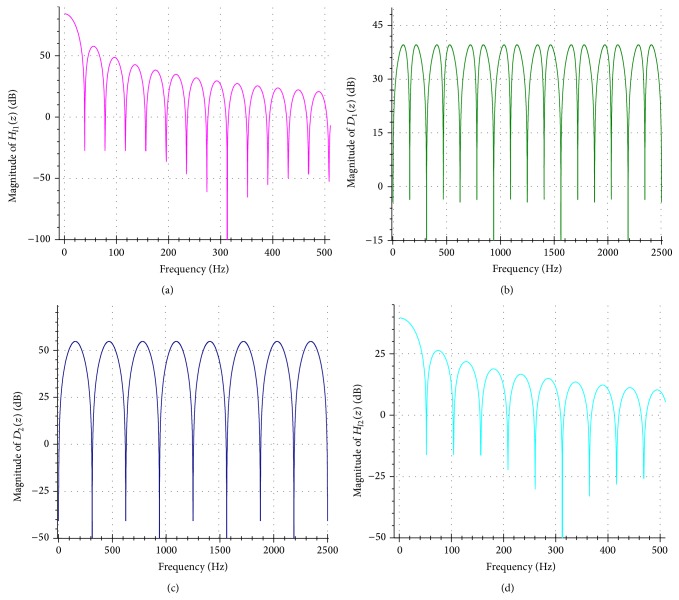
Frequency responses of the employed digital filters in the present study. (a) Frequency response of *H*_*l*1_(*z*). (b) Frequency response of *D*_1_(*z*). (c) Frequency response of *D*_2_(*z*). (d) Frequency response of *H*_*l*2_(*z*). The sampling rate is 5000 Hz.

A second-order, linear phase, low-pass filter is employed for suppressing high-frequency noise that might contaminate the pulse signal. The transfer function of this filter is given by

(5) $$\begin{array}{c} H_{l1}\left(z\right)=\frac{1-2z^{-128}+z^{-256}}{1-2z^{-1}+z^{-2}}, \end{array}$$

where the gain is Gain1 = 16384 = 2^14^ and the intrinsic delay of *H*_*l*1_(*z*) is 127 samples.  Figure 2(a) displays the frequency response of this filter. The utilization of *H*_*l*1_(*z*) provides a main benefit: its linear-phase characteristic leaves the phase of the frequency components lying within the pass band of this filter undistorted. Let *x*[*n*] denote the input raw signal and let *y*[*n*] denote the output signal. Figures 1(b) and 1(c) illustrate the filtered signals obtained from brachial and radial arteries, respectively.

#### 2.3.2. Differential Filters

The differentiation technique is widely used as a tool for extracting valuable information about the physiologic process contained in the physiological signal. Regarding the pulse waveforms, the elaborately designed differential filters can provide an advantage in reducing the low-frequency interferences, such as baseline drifting and respiratory fluctuations.

*(i) The First-Order Differential Filter*. A 7-point stencil first-order, central differential filter is applied to the output (i.e., *y*[*n*]) of *H*_*l*1_(*z*) [30]:

(6) $$\begin{array}{c} D_{1}\left(z\right)=-\left(1-9z^{-16}+45z^{-32}-45z^{-64}+9z^{-80}-z^{-96}\right), \end{array}$$

where the gain is Gain2 = 960 and the intrinsic delay of *D*_1_(*z*) is 48 samples.  Figure 2(b) shows the frequency response of this filter. Let *d*1[*n*] denote the output of this filter. 

*(ii) The Second-Order Differential Filter*. The decision rules based on the second-order differential filter for feature extraction are also adopted in the literature. To identify the performance of this kind of methods, likewise, a 7-point central differential filter is applied to the previously obtained *y*[*n*] [30]:

(7) D2z=1−13.5z−16+135z−32−245z−48 + 135z−64−13.5z−80+z−96,

where the gain is Gain3 = 23040 and the intrinsic delay of *D*_2_(*z*) is 48 samples. Let *d*2[*n*] be the output of this filter.  Figure 2(c) depicts the frequency response of *D*_2_(*z*). 

*(iii) The Low-Pass Filter*. To further suppress the high-frequency noise caused by the differential filters *D*_1_(*z*) and *D*_2_(*z*), we smooth the resultant *d*1[*n*] and *d*2[*n*] with a simple integer and low-pass filter of the form in (8), respectively;

(8) $$\begin{array}{c} H_{l2}\left(z\right)=\frac{1-z^{-96}}{1-z^{-1}}, \end{array}$$

where the gain is Gain4 = 96 and the intrinsic delay of *H*_*l*2_(*z*) is 47.5 samples.  Figure 2(d) delineates the frequency response of this filter. Let *d*1′[*n*] and *d*2′[*n*] denote the corresponding output signals of *H*_*l*2_(*z*), respectively. Figure 1(d) is an example that displays two channels of *d*1′[*n*] by using the filters *D*_1_(*z*) and *H*_*l*2_(*z*). Figure 1(e) illustrates two channels of *d*2′[*n*] by using the filters *D*_2_(*z*) and *H*_*l*2_(*z*). 

*(iv) Slope Sum Function*. The slope sum function (SSF) technique was firstly introduced in [31]. A similar form (9) is proposed in the present design but is modified to accommodate the different sampling rate of the pulse data (i.e., 5000 Hz):

(9) w[n]=1N∑k=0N−1Δμ[n∗],Δμ[n∗]=Δy[n∗],if  Δy[n∗]>0,0,if  Δy[n∗]⩽0,

where *N* = 96, *n* is the sample number, and *n* ^*^ = *n* − *k*. Δ*y*[*n*] = −*y*[*n* − 48] + 9*y*[*n* − 32] − 45*y*[*n* − 16] + 45*y*[*n* + 16] − 9*y*[*n* + 32] + *y*[*n* + 48]. Where, *y*[*n*] represents the output of *H*_*l*1_(*z*). It is apparent that the SSF defined in (9) is equivalent to that first let *tm*[*n*] = *d*1[*n*], if *tm*[*n*] < 0, let *tm*[*n*] = 0; then make *tm*[*n*] be applied to the filter *H*_*l*2_(*z*), and the ultimate output is *w*[*n*]. The inset Figure 1(f) demonstrates the SSF signals of two channels.

### 2.4. Pulse-Beat Detection

Prior to the determination of the reference feature point on the single waveform, each pulse beat should be detected. Because the pulse beat can be readily identified, we briefly outline the algorithm here, and full details are beyond the objective of this paper. It is based on a simplification of Pan-Tompkins algorithm which is widely used for QRS detection in electrocardiogram [32]. Its procedures are as follows. 

*(1) Extraction of Variation Information*. The two channel raw signals are both applied to a differential filter 1 − *z* ^−128^; we then obtain corresponding sequences of the variation information of the raw signals. 

*(2) Squaring Operation*. Subsequently, a nonlinear transformation is performed which consists of point by point squaring of the samples in the sequences obtained from* (1)*. It aims to amplify the high-frequency information of raw signals. 

*(3) Threshold Definition*. Two automatically update threshold values can be determined by processing the sequences obtained from* (2)* using a simple low-pass filter (1 − *z* ^−16384^)/(1 − *z* ^−1^). The gain of this filter is 16384. The samples of two sequences in* (2)* are synchronously divided by these two thresholds in real time, respectively. We then obtain two new sequences. It is apparent that* (3)* is similar to a normalization process. 

*(4) Moving Window Integration*. We then construct a new sequence by the sum operation of two sequences obtained from* (3)*. The integration operation is taken over the constructed sequence with a moving window of the width of 450 points, which is accomplished by an integer digital filter (1 − *z* ^−450^)/(1 − *z* ^−1^). The gain of the filer is 450. The utilization of this filter is to attenuate the higher frequencies associated with the device noise. 

*(5) Pulse-Beat Detection*. Similar to* (3)*, a basic threshold value is calculated by processing the sequence in* (4)* with the aforementioned filter (1 − *z* ^−16384^)/(1 − *z* ^−1^), and a decisive threshold value is also defined as two times of the basic threshold. A buffer array is used to record the samples in this sequence if the samples are larger than the basic threshold. Therefore, the data buffer is now scanned by counting the data points which meet or exceed the decisive threshold. If 200 or more of these points in this buffer meet or exceed the threshold, then the data buffer is associated with a pulse beat.

### 2.5. Feature Extraction Based on Different Decision Rules

**Figure 3:**
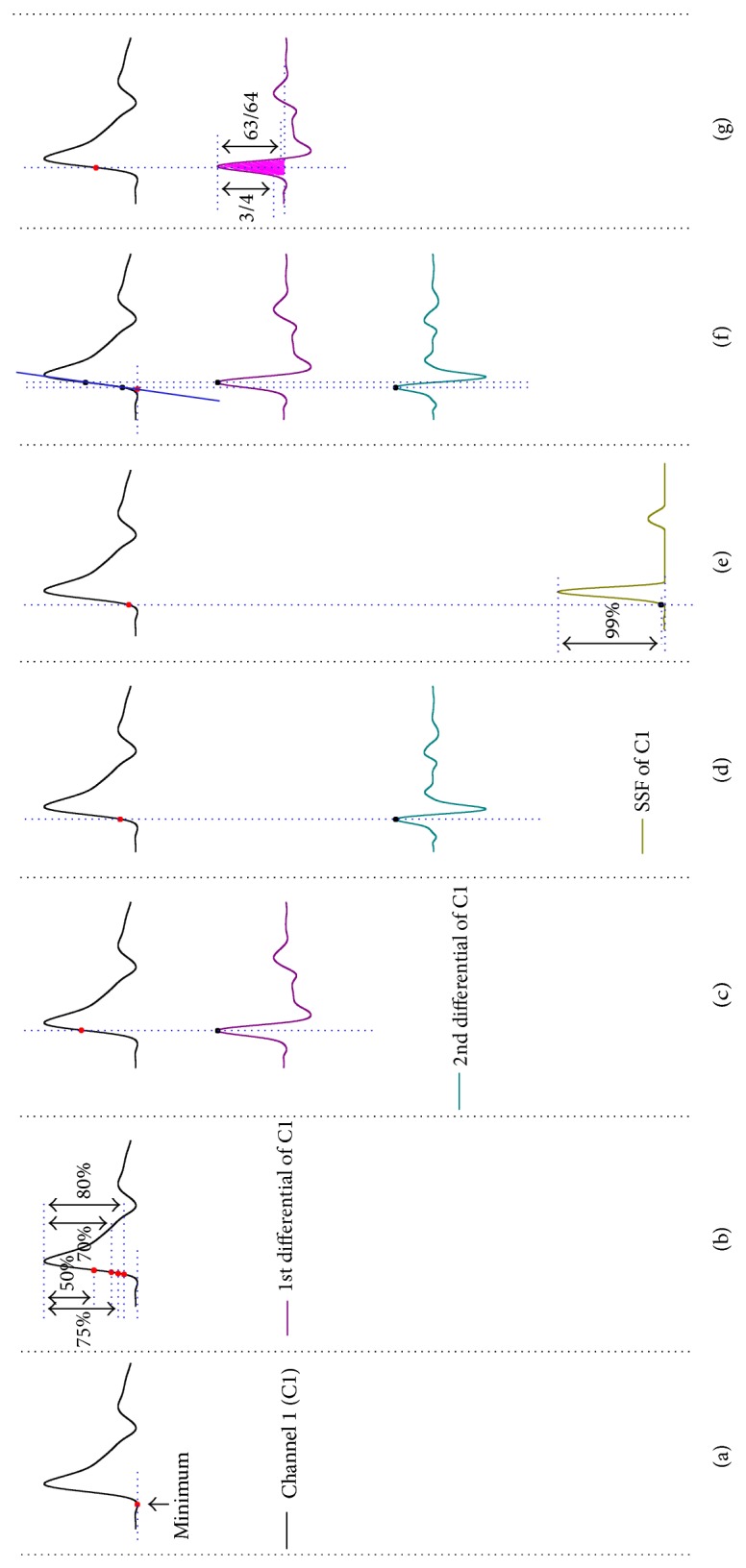
The determination of feature point for the PTT estimation using different methods. (a) MIN method. (b) Direct threshold methods (TH20%, TH25%, TH30%, and TH50%). (c) D1 method. (d) D2 method. (e) SSF method. (f) Transect intersection methods (TAN1 and TAN2). (g) McM method. See the text for abbreviation.

In the present study, we investigated the efficacy of 10 different computerized methods, as well as a newly proposed method for the determination of the reference point on the pulse waveform. For each detected pulse beat, Figure 3 presents how the feature point is determined by 11 methods which are briefly described as follows. 

*(i) Minimum of the Pulse Wave*. It is defined as the minimum point of the segment lying in the trough area of two adjacent pulse waves (MIN, the offset of the first wave in diastolic phase and the onset of the next wave in systolic phase) [10, 15, 27], as depicted in Figure 3(a). It can be determined by a threshold-slope technique which is introduced in [10]. 

*(ii) Direct Threshold Methods*. They seek out the reference point as the last point moving from the minimum point with an intensity value less than a specified threshold before the systolic peak of the current pulse waveform, as rendered in Figure 3(b). The thresholds are set at 0.2 [19], 0.25 [11, 20], 0.30 [18], and 0.50 [12, 13, 15] times of the pulse pressure above the diastolic pressure (i.e., the trough-to-peak height), and these methods are denoted as TH20%, TH25%, TH30%, and TH50%, respectively. 

*(iii) Peak of the First-Order Derivative [10, 14, 15, 17]*. In *d*1′[*n*], we determine the maximum of the *d*1′[*n*] in the whole duration of the pulse as the feature point. It is apparent that this reference point is closest to the median position in the systolic phase of each pulse waveform; see Figure 3(c) for clarification. 

*(iv) Peak of the Second-Order Derivative [10, 15, 19]*. In *d*2′[*n*], we determine the maximum of the *d*2′[*n*] in the whole duration of the pulse as the feature point. This reference point is closest to the minimum (i.e., onset of systolic wave) of the systolic phase of each pulse waveform. Figure 3(d) portrays this. 

*(v) SSF Method*. Recalling the sequence *w*[*n*] obtained by the operation (9), the SSF defines the point as the reference point at which the resultant *w*[*n*] reaches a threshold, as can be seen in Figure 3(e). The threshold level for this method is counted as a fraction of the amplitude of the peak of *w*[*n*] in the corresponding pulse waveform, and the threshold level is 1.0% [31]. 

*(vi) Transect Intersection Methods*. Two transect intersection methods (denoted as TAN1 and TAN2, resp.) are investigated here. TAN1 begins by generating a line that passes through two positions of pulse signal *y*[*n*] which are corresponding to the peaks of the first-order derivative in (iii) and the second-order derivative in (iv), respectively; the reference point is determined by the intersection of this line and the horizontal line that passes through the minimum point obtained in (i) [18]. TAN2 is used by producing a line that is centered at the site of the signal *y*[*n*] which is related to the previously determined peak of the first-order derivative in (iii). Data points are then synchronously added on both forward and backward sides of this site until the CC between the data set of *y*[*n*] and the set of the data generated by least-square fitting is less than 0.999 [10]. Likewise, the reference point is specified as the intersection of the line fitted by the just generated data and the horizontal line that passes through the minimum point obtained in (i). Inspect Figure 3(f) for details. 

*(vii) Method Based on the Centroid Method (McM)*. We first obtain the point with maximum value of the first-order derivative in the current area using (iii) on *d*1′[*n*]. In the leftward direction of this peak, we thus can determine a point at which the amplitude is less than 1/4 of this peak. In the rightward direction, we can also determine another point at which the amplitude is less than 1/64 of this peak. Figure 3(g) reveals this process. The introduction of threshold values brings about two advantages: the use of the first threshold (i.e., 1/4) could reduce the fluctuation interference caused by noise source around the onset of the systolic phase, and the use of the second threshold (i.e., 1/64) could lessen morphological variations which are mostly caused by predicrotic and dicrotic waves. With these points between two reference positions, we then determine the optimal feature point based on the McM:

(10) $$\begin{array}{c} R_{c}\left[m_{c}\right]=\frac{\sum d1^{\prime }\left[i\right]r\left[i\right]}{\sum d1^{\prime }\left[i\right]}, \end{array}$$

where *i* is the index for the points lying within these two reference positions and *R*_*c*_[*i*] is the distance from the leftward reference point to the *i*-index sample. *m*_*c*_ is optimal point obtained. Because the differential filter *D*_1_(*z*) is sensitive to high-frequency noise, the application of (10) can significantly eliminate this kind of noise. We will quantitatively evaluate it in the next section.

We repeat (i) to (vii) for determining the feature points for each paired pulse beats in two channel signals, respectively. Therefore, we can obtain 11 PTTs for each cardiac beat.

## 3. Results and Discussion

### 3.1. Results of Artificial Pulse Data Sets

Because it is difficult to accurately estimate the absolute value of PTT, the artificial data are generated to investigate the reproducibilities (i.e., mean ± standard deviation [SD]) of all decision rules introduced in this study. As aforementioned, for each in four publicly available artificial records [28], we constructed two 1800-second duration sequences and resampled them at 5000 Hz, including about 1800 paired pulse beats, and made one have a time delay of 250 ms by the circular-shifting operation. Thereby, for each paired pulse waves in the constructed sequences, they had a distance with 250 ms, and this distance was regarded as the PTT duration. By this way, we obtained 4 paired pulse sequences with the PTT duration of 250 ms.

**Figure 4:**
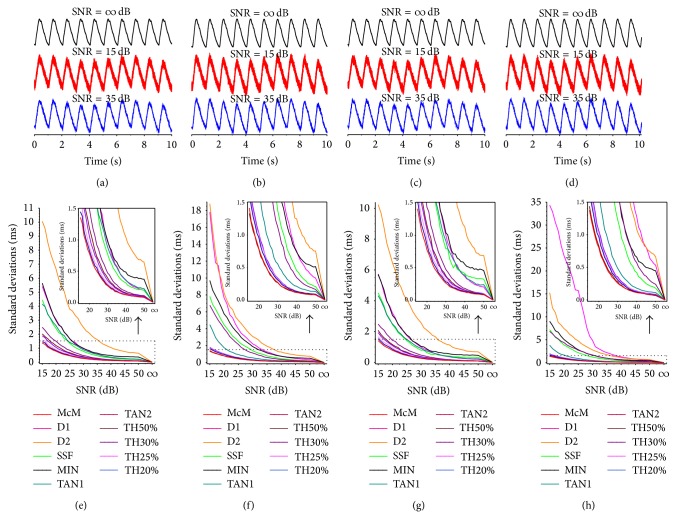
Statistical results of the artificial data with various noise levels using different decision rules. From left to right: insets (a) to (d) show 4 artificial data with the addition of various intensity of simulated noise (i.e., SNR = *∞*, 15, and 35 dB, resp.). (e) to (h) show the distributions of standard deviations of all 11 methods.

A major purpose for the utilization of artificial pulse data is to assess the newly proposed approach and the traditional methods in a noisy environment. The zero-mean, Gaussian white noise were added to each paired sequences data to reproduce artificial pulse waves with SNR from 15 to 50 dB, in increments of 1 dB (note that the Gaussian white noise in each constructed sequence is different from each other), as well as a *∞* dB case. For each specified SNR case, the noisy data was then mixed with artificial respiratory signal which was generated by a cosine function with the amplitude of a tenth of the simulated pulse wave amplitude and the intrinsic frequency of 1/6 Hz (i.e., 10 beats per minute). We thus obtained 37 sets of paired data for 4 constructed sequences. Figures 4(a) to 4(d) show the simulated pulse data with SNR of *∞*, 15, and 35 dB for constructed sequences, respectively.

The performance of each method was summarized as the mean and SD of the bias and precision values across all the simulated pulse data at various SNR levels. For each method, the mean is calculated by averaging distances of the feature points measured from every paired pulse waves in each paired constructed sequences. The SD of distances is also calculated to investigate the dispersion from the mean at a specified SNR case. The mean differences are less than 1 ms for all methods. Figures 4(e) to 4(h) only delineate the SD distributions with respect to different SNR values for 4 kind constructed sequences for all methods, respectively. From Figures 4(e) to 4(h), for the SNR = *∞* dB case, it is clear that the SDs are all equal to 0 for all of these methods, which implies that the reproducibility for each method at SNR = *∞* dB is 100%. However, for various SNR values, the performances of different methods are significantly different. In accordance with the distributions of SDs in Figures 4(e) to 4(h), collectively, we conclude that when SNR → *∞* dB, then SD → 0 ms for all of these methods, and they coincide with practical situations. It is observed that the MIN, SSF, D1, and D2 methods have the poorest performances since they are extremely sensitive to high-frequency noise interference; the least-square technique based TAN1 and TAN2 methods demonstrate modest performances; the direct threshold methods (i.e., TH20%, TH25%, TH30%, and TH50%) show favorable agreement and better performances. Regarding the accuracy as a whole, the McM method outperforms the other 10 methods: most of the SDs are less than 1 ms for various SNR cases.

### 3.2. Results of Clinical Pulse Data Sets

The absolute PTT values obtained from various measurement methods are often not interchangeable when in fact different methods might cause substantially different PTT values for the clinical pulse data. The relationship between values of beat-to-beat BP alteration and the corresponding values of beat-to-beat PTT variation, that is, (4), is thus applied for investigation with clinical data.

For each method, the PTT is measured in real time as the time interval from the brachial wave to the radial wave for each paired waves. For all of these methods, we thus can obtain 33 × 11 sequences of PTT data (denoted as *T* for each sequence as before) and 33 × 11 sequences of BP data (also denoted as *P* for each sequence as before). We first let all of these PTT and BP sequences be processed with a median filter with the window of a width of 31 points. This median filter is designed to eliminate the artificial outliers as well as removing the very low-frequency drifting caused by respiratory fluctuation. Let $\bar{T}$ and $\bar{P}$ be the outputs of *T* and *P* of the nonlinear filter, respectively. Thereafter, let the sequences $\bar{P}$ be applied to a simple forward derivative as below to obtain the variation of BP (denoted as Δ*P* for each record):

(11) $$\begin{array}{c} \begin{array}{c} \Delta P\left[n\right]=\bar{P}\left[n\right]-\bar{P}\left[n-31\right] \end{array}. \end{array}$$

And then, the corresponding synchronous variation of PTT can be counted according to (12) (denoted as Δ*T*/*T* for each record):

(12) $$\begin{array}{c} \frac{\Delta T\left[n\right]}{T\left[n\right]}=\overset{n}{\underset{i=n-29}{\sum }}\frac{\bar{T}\left[i\right]-\bar{T}\left[i^{*}\right]}{\bar{T}\left[i^{*}\right]}, \end{array}$$

where *i* ^*^ = *i* − 1. The relationships of sequences of Δ*P* and −Δ*T*/*T* are then quantitatively examined (hereinafter, Δ*P* and −Δ*T*/*T* denote two sequences, resp.).

**Figure 5:**
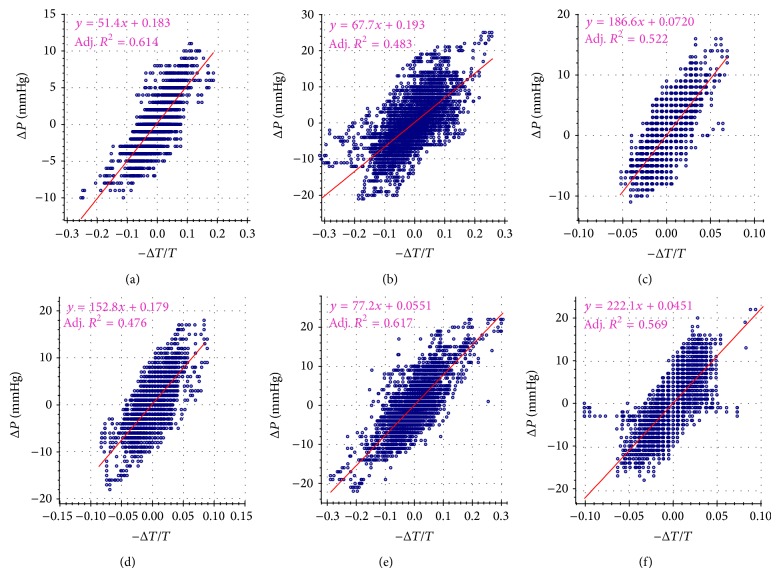
Linear regression analysis of Δ*P* and −Δ*T*/*T* relationships in 6 typical subjects which are indexed as (a) to (f), respectively. The linear-fitting results are listed on the top left corner in each inset.

According to the basic formula (4), for the results obtained from each participant using the McM method, we then plotted the obtained Δ*P* against −Δ*T*/*T* in scatter way.  Figure 5 shows the corresponding results of 6 typical participants, in which 6 subfigures depict results of 6 subjects, respectively. The red lines are linear least-squares fit of Δ*P*[*n*] with respect to −Δ*T*[*n*]/*T*[*n*]. From Figure 5, it can be apparently seen that the adjusted R-squared coefficients of determination fall within [0.476, 0.617] and the intercept values are less than 0.2 mmHg, which indicate that there is a reasonably good agreement with (4).

**Figure 6:**
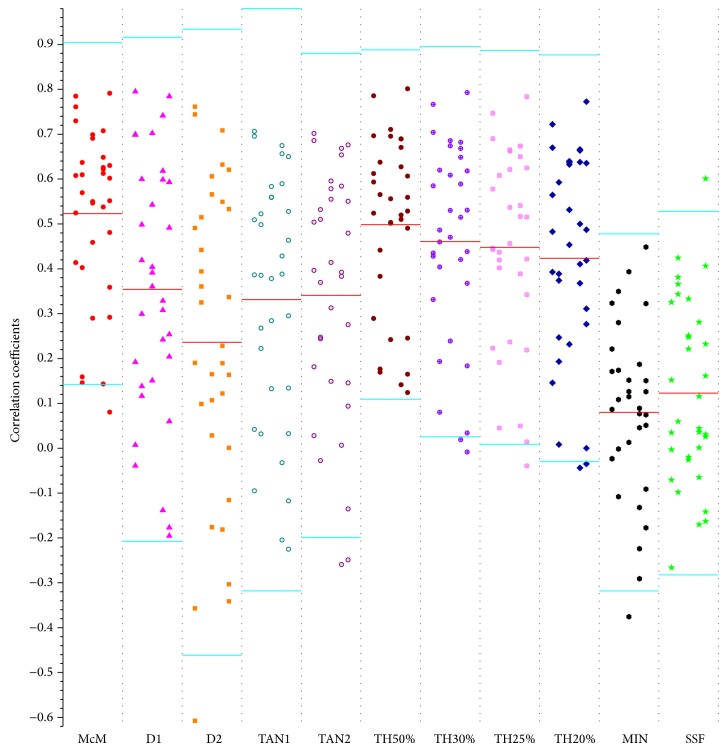
Test results of clinical pulse data obtained from all of these methods for 33 participators. Each column contains a distribution of 33 CCs calculated from all the subjects for a specific method. In each column, the red line indicates mean values of CCs, and the cyan lines represent the 95% confidence interval of CCs.

For each subject, the CC between Δ*P* and −Δ*T*/*T* is calculated using each method. We can thus obtain 33 CCs for each method. The 95% of confidence interval (i.e., mean ± 1.96 SD) of CCs were also calculated. In Figure 6, each column contains the 33 CCs counted by a specified method. In each colum, the mean and 95% of confidence interval were signed in red and cyan lines, respectively.  Table 1 depicts the summary statistics of all the CCs for these methods.

In terms of the MIN method, from the tenth column of Figure 6 and the second row of Table 1, we see that all CCs are less than 0.45 and most CCs fall in [0.1, 0.2]; several negative CCs are also observed, and the median CC is 0.08900. When comparing the CCs of this method with these of the McM method, in fact, there are only 2 CCs larger than those of the McM method. Because the MIN method is vulnerable to high- and low-frequency fluctuations around the same area, all of these descriptions confirmed the poor performance of the MIN method.

With regard to the D1, D2, and SSF methods, from Figure 6 (the second, third, and last columns) and the Table 1, we note that the D1 and D2 methods show similar performances. Statistically, the performance of D1 method is slightly better than that of D2 method (from Table 1, the Mean ± SDs are 0.3542 ± 0.2866 and 0.2362 ± 0.3560 for D1 and D2 methods, resp.). This indicates that the D2 method is more vulnerable to the high-frequency noise interference. For the SSF method, most of CCs are less than 0.4 as we can see in the last column of Figure 6. Recalling Figure 6 and Table 1, we can conclude that its performance is poorer than that of D1 and D2 methods since the determination of the peak of SSF coefficients is required, which is quite sensitive to wide-band noise.

The straightforward threshold-based methods (i.e., TH20%, TH25%, TH30%, and TH50%) produce CCs on the order of 0.41 ~ 0.49. Overall, from Figure 6 (six to ninth columns) and Table 1, it is interesting that the four methods perform quite similarly, possibly because of the similar capabilities of these methods for interference elimination. Collectively, the higher the threshold the slightly better the performance: from Table 1, the Mean ± SDs are 0.4235 ± 0.2312, 0.4479 ± 0.0.2239, 0.4605 ± 0.0.2219, and 0.4985 ± 0.1987 for TH20%, TH25%, TH30%, and TH50%, respectively. Compared with other existing methods, the TH50% shows a closest performance to that of the McM method.

From Figure 6 (the fourth and fifth columns) and the Table 1, concerning the transect intersection methods (i.e., TAN1 and TAN2). We note that there is no significant difference in performance. In Table 1, the Mean ± SDs are 0.3311 ± 0.2783 and 0.3407 ± 0.2754, respectively. The median values of CCs are 0.3862 and 0.3921, respectively. Because the only difference of these two methods is the tactics that different techniques are applied for determining the transect line and this indirectly means that different rules for determining the transect line make no apparent contribution to the performance. A possible explanation for the observed relatively poorer performance might be that performance of these types of methods is more tightly related to the feature point obtained by the MIN method.

Performance of this newly proposed McM method can be deduced from the first column of Figure 6 and first row of Table 1; it is clear that most of the CCs fall within [0.5, 0.7] as we can apparently see from Figure 6. Of note, in contrast to other 10 candidate methods summarized in Table 1, in the total study population, the McM method has the maximum values of mean (0.5231), sum (17.26), and median (0.5695) and has the minimum value of SD (0.1943), which, together with referring to other columns in Figure 6, indicate that the McM method has the best performance.

### 3.3. Benefits and Limitations

It is worth emphasizing that test results of both artificial and clinical pulse data using all methods exhibit overall performance tendencies which imply that the two metrics for evaluation mutually validate the feasibility. These test results for both data sets reveal that the MIN method is inappropriate for determining feature point in PTT measurement because of its high bias, high dispersion (i.e., large SDs), and low CCs. In effect, the minimum point of pulse wave is quite gradual at the diastolic branch for some clinical data. Overall, the results of this study demonstrate that the combination of differential method, double-threshold settings, and the centroid method yields a reliable method for PTT estimation. This new method exhibits a higher detection rate than previous decision rules. This could possibly lead to incorporation into pulse-wave detection devices to improve the reproducibility and accuracy.

Accurate measurement of PTT might provide help for robust BP estimation diagnosis of CVDs, the early prediction of CVDs, and so on. In the present study, both artificial and clinical pulse data are used for assessing the existing decision rules and a newly presented method McM. The results of artificial data at different SNR levels demonstrated that the McM method has the best reproducibility. A clinical population of 33 participants is used for investigation based on the Moens-Korteweg equation. A few low positive CCs have been observed for each method. In spite of this, the statistical results confirmed that the McM method, collectively, has the best performance which indicates it would be readily utilized in routine clinical practice for risk stratification, prognosis, and cost-effective preventive therapy.

We can speculate that robust determination of PTT (*T*) would yield an accurate measurement of relative variation of PTT (Δ*T*/*T*). In the meanwhile, we acknowledge that reliable estimation of Δ*T*/*T* does not definitely mean that we could obtain a reliable detection of the absolute PTT duration. Because it is difficult to obtain the true duration of PTT in vivo, the Moens-Korteweg equation, that is, (1), is then employed for evaluation in the current study. The application of (1) is based on some assumptions including that the vessel wall is isotropic [33]. Human's blood vessels are far more complicated than an ideal elastic tube. In addition, (4) is an approximate estimate of relationship between the Δ*P* and Δ*T*/*T*, and several associated factors might need to be considered for investigation. Reciprocally, several results of low positive CCs observed in this study may provide additional insight regarding (4) which should be further investigated [27]. Nevertheless, it is thus an inherent limitation of our study.

As mentioned in the Introduction section, various methods for measurement of the PTT have been developed in the literature, 10 candidate methods are briefly reviewed and investigated in this study. Noteworthily, the utilization of different decision rules might adopt different digital signal processing (DSP) techniques proposed by different research groups. In addition, several technical specifications of the DSP algorithms are unavailable or have not been disclosed for some existing methods and especially for the methods proposed by commercial manufacturers. We thus only assessed the decision rules for feature-point localization on the pulse waves via the same DSP technique. It possibly implies that poor performance of the existing decision rules evaluated here does not mean that the original methods proposed by previous investigators have poor performance. In this regard, it is another weakness of our study.

## 4. Conclusions

Accurate identification of the fiducial point of the pulse waveform is deemed essential for the estimation of PTT duration. Thus far, there is no well-established method for PTT measurement. In the present study, firstly, we introduced the Moens-Korteweg equation and its deduced equation for investigating the relationship of PTT and BP. A series of integer digital filters are subsequently designed for pulse signal processing. Several existing decision rules for feature-point localization have been briefly reviewed, and a novel approach which consists of the differential method, threshold method, and the centroid method is proposed. Ultimately, the new and existing methods are investigated with both artificial and clinical pulse data. Taken together, our evaluation results of artificial and clinical data indicate that this newly presented method outperforms the existing methods. Nonetheless, it is important to note that, even for this newly proposed method, several low CCs of clinical data have been observed. From the standpoints of biosignal detection and hemodynamics, this method remains to be optimized on larger samples. Whereas those low positive CCs are of clinical interest and would be worthy of systematic investigation in examining whether the variation of BP (Δ*P*) is a principal or modest determinant of the relative change of PTT (Δ*T*/*T*).

## Figures and Tables

**Table 1:** Statistical analysis of correlations between Δ*P* and −Δ*T*/*T* calculated from all candidate methods for 33 participators.

Methods	Mean	SD^‡^	Sum	Minimum	Median	Maximum
McM	0.5231	0.1943	17.26	0.08030	0.5695	0.7913
MIN	0.07990	0.2011	2.637	−0.3756	0.08900	0.4484
D1	0.3542	0.2866	11.69	−0.1955	0.3605	0.7950
D2	0.2362	0.3560	7.793	−0.6076	0.2283	0.7611
SSF	0.1229	0.2067	4.054	−0.2660	0.05950	0.6010
Th50%	0.4985	0.1987	16.45	0.1241	0.5285	0.8013
Th30%	0.4605	0.2219	15.20	−0.008500	0.4862	0.7926
Th25%	0.4479	0.2239	14.78	−0.03950	0.4564	0.7834
Th20%	0.4235	0.2312	13.98	−0.04390	0.4534	0.7727
TAN1	0.3311	0.2783	10.93	−0.2253	0.3862	0.7064
TAN2	0.3407	0.2754	11.24	−0.2594	0.3921	0.7016

^‡^indicates standard deviation. See text for abbreviation.
